# Cocaine Self-Administration Increases Impulsive Decision-Making in Low-Impulsive Rats Associated with Impaired Functional Connectivity in the Mesocorticolimbic System

**DOI:** 10.1523/ENEURO.0408-24.2025

**Published:** 2025-03-11

**Authors:** Hui Shen, Zilu Ma, Emma Hans, Ying Duan, Guo-Hua Bi, Yurim C. Chae, Robbie Y. Kuang, Zheng-Xiong Xi, Yihong Yang

**Affiliations:** ^1^Neuroimaging Research Branch, National Institute on Drug Abuse, Intramural Research Program, Baltimore, Maryland 21224; ^2^Addiction Biology Unit, Molecular Targets and Medications Discovery Branch, National Institute on Drug Abuse, Intramural Research Program, Baltimore, Maryland 21224

**Keywords:** choice impulsivity, cocaine self-administration, dopamine receptors, functional connectivity, functional MRI, reward delay discounting

## Abstract

Impulsivity is often considered a risk factor for drug addiction; however, not all evidence supports this view. In the present study, we used a food reward delay-discounting task (DDT) to categorize rats as low-, middle-, and high-impulsive but failed to find any difference among these groups in the acquisition and maintenance of cocaine self-administration (SA), regardless of electrical footshock punishment. Additionally, there were no group differences in locomotor responses to acute cocaine in rats with or without a history of cocaine SA. Unexpectedly, chronic cocaine SA selectively increased impulsive choice in low-impulsive rats. Resting-state fMRI analysis revealed a positive correlation between impulsivity and cerebral blood volume in the midbrain, thalamus, and auditory cortex. Using these three regions as seeds, we observed a negative correlation between impulsivity and functional connectivity between the midbrain and frontal cortex, as well as between the thalamus and frontal cortex (including the orbitofrontal, primary, and parietal cortices) in low-impulsive rats. These correlations were attenuated following chronic cocaine SA. RNAscope in situ hybridization assays revealed a significant reduction in dopamine (DA) D_1_, D_2_, and D_3_ receptor mRNA expression in the corticostriatal regions of low-impulsive rats after cocaine SA. Our findings challenge the widely held view that impulsivity is a vulnerability factor for cocaine use disorder. Instead, chronic cocaine use appears to selectively increase impulsive choice decision-making in normally low-impulsive rats, associated with reduced functional connectivity and DA receptor expression in the mesocorticolimbic DA network.

## Significance Statement

Impulsivity has long been considered a risk factor for substance use disorders (SUDs). However, findings across different impulsivity measures have been inconsistent or controversial. In this study, we did not find evidence supporting the notion that preexisting choice impulsivity is a predictive factor for compulsive cocaine self-administration (SA). Instead, we found that chronic cocaine SA led to a significant increase in impulsive choice decision-making in normally low-impulsive rats. This increase was associated with reduced functional connectivity and reduced dopamine receptor expression in the dopamine-related network. Our findings suggest that choice impulsivity does not predict SUD; rather, chronic cocaine use is a risk factor for developing impulsive behavior in healthy individuals.

## Introduction

Substance use disorder (SUD) is characterized by the uncontrollable use of a substance despite harmful consequences (First, 2013). Identifying vulnerability factors for SUD is a major challenge in drug abuse research. Impulsivity has long been thought as a risk factor for SUD (Dalley et al., 2007, 2008; Weafer et al., 2014; Dalley and Ersche, 2019).

Impulsivity is often classified as impulsive action (inability to stop a premature response) and impulsive choice. Impulsive choice can further be divided into risk- and delay-discounting choice (Winstanley et al., 2006; Jentsch et al., 2014). In risky choice tasks, animals choose between a small, “safe” (100% certain) food reward and a larger, “risky” reward with either a lower probability of obtaining the reward or an increasing probability of a mild footshock punishment (Dalley and Robbins, 2017; Orsini et al., 2020). Delay-discounting impulsive choice is often measured using delay-discounting tasks (DDT), where impulsive individuals prefer smaller, immediate rewards over larger, delayed ones. Impulsive action is often measured using the 5-choice serial reaction time (5CSRT) task (Dalley and Robbins, 2017). Studies have shown that high impulsive action and risk impulsive choice are prevalent in patients with SUD (Bechara et al., 2002; Verdejo-Garcia et al., 2007; Ersche et al., 2010; Jentsch et al., 2014). However, not all clinical evidence supports this observation. For example, higher impulsive decision-making is more often seen in cocaine users than heroin users (Bornovalova et al., 2005). Long-term amphetamine users show impaired decision-making, while opiate users do not (Rogers et al., 1999).

Preclinical findings on the predictive value of impulsivity for drug abuse are also mixed. High-impulsive rats showed higher cocaine intake (Perry et al., 2005; Dalley et al., 2007; Orsini et al., 2020; Urueña-Méndez et al., 2023), higher break-point levels under progressive-ratio schedules for cocaine, persistent responding for cocaine in the presence of electrical footshock (Belin et al., 2008), and higher levels of reinstatement responding (Perry et al., 2008; Economidou et al., 2009; Broos et al., 2012). These findings suggest that high impulsivity may predict the switch to compulsive drug-taking and drug-seeking behavior.

However, not all evidence supports these observations. Paradoxically, findings from the same reports also showed no difference in cocaine self-administration (SA) between high- and low-impulsive rats in either 5CSRT (Belin et al., 2008; Caprioli et al., 2013; Abbott et al., 2022), risky choice task under short-access conditions (Orsini et al., 2020), or DDT (Perry et al., 2008). In contrast, low-impulsive or low-risky female rats took more cocaine than high-impulsive or high-risky counterparts under short-access conditions (Perry et al., 2008; Orsini et al., 2020). Furthermore, risky impulsive choice predicts cocaine intake in adolescent, not in adult rats (Mitchell et al., 2014a). In adults, risky decision-making might be associated with incubation of craving, but not drug intake (Ferland and Winstanley, 2017). There is no difference in progressive-ratio break-point levels for cocaine SA between high-impulsive Roman high-avoidance rats and low-impulsive control rats (Arrondeau et al., 2023). These conflicting findings suggest that different dimensions or subtypes of impulsivity may have distinct roles in drug-taking and drug-seeking behaviors.

In addition to the predictive value of impulsivity in drug abuse, findings on the effects of chronic drug use on impulsive behaviors are also mixed. Studies have reported both decreases and increases in impulsive action following cocaine use, as assessed by premature responding on the 5-CSRT task (Dalley et al., 2007; Winstanley et al., 2009; Caprioli et al., 2013), while other studies showed no alterations in impulsivity after cocaine withdrawal (Dalley et al., 2005; Ferland and Winstanley, 2017; Abbott et al., 2022; Urueña-Méndez et al., 2023). These inconsistencies highlight the need for further studies to understand the causal relationship between impulsivity and loss of control over drug use.

While most studies used 5CSRT and risk choice tasks, fewer studies used DDT to explore the causal relationship between choice impulsivity and SUD. In this study, we aimed to determine: (1) whether high- and low-impulsive rats differ in cocaine SA; (2) whether chronic cocaine SA alters impulsive choice decision-making; and (3) whether cocaine-induced changes in impulsivity are associated with alterations in brain functional connectivity and DA receptor expression. Our results showed no difference in either cocaine SA or locomotor response to cocaine between high- and low-impulsive rats. Unexpectedly, chronic cocaine SA increased impulsivity only in normally low-impulsive rats, which is linked to attenuated functional connectivity in the midbrain-frontal and thalamus-frontal circuits and reduced DA receptor expression in the corticostriatal regions observed in this group of rats.

## Materials and Methods

### Animals

A total of 105 male Long–Evans rats (250–350 g) from Charles River Laboratories were used in the present study. Among them, 38 rats had cocaine SA (of which 24 rats underwent fMRI scans), 13 rats had sucrose SA as control, and 54 rats were used for RNAscope ISH and open-field locomotion experiments. The male rats were chosen as significant gender differences exist between males and females in impulsive decision-making (Silverman, 2003; Perry et al., 2005; Myerson et al., 2015; Hammerslag et al., 2019). Rats were individually housed in a climate-controlled colony room and maintained on a 12 h reversed light/dark cycle (lights on at 7:00 P.M., lights off at 7:00 A.M.) with water *ad libitum* in their home cages. Rats were placed on food restriction for at least 1 week prior to behavioral training and were maintained at ∼85% of free-feeding weight for the duration of acquisition training and delay-discounting task schedule. Animals were weighed daily and fed 12 g of chow per day. All experiments were approved by the Animal Care and Use Committee of the National Institute on Drug Abuse of the National Institutes of Health.

### Experiment 1: reward delay discounting

#### Apparatus

Behavioral experimental sessions were conducted in 15 operant test chambers (30.5 × 24.1 × 21.0 cm) from Med Associates. Each chamber contained a food pellet dispenser and receptacle, located between two retractable, 28 V light cue-associated response levers located 6.5 cm from the test chamber floor. A 28 V house light remained illuminated throughout sessions and a 2,900 Hz sound cue was delivered following each lever press.

#### Procedures

**Figure 1.**
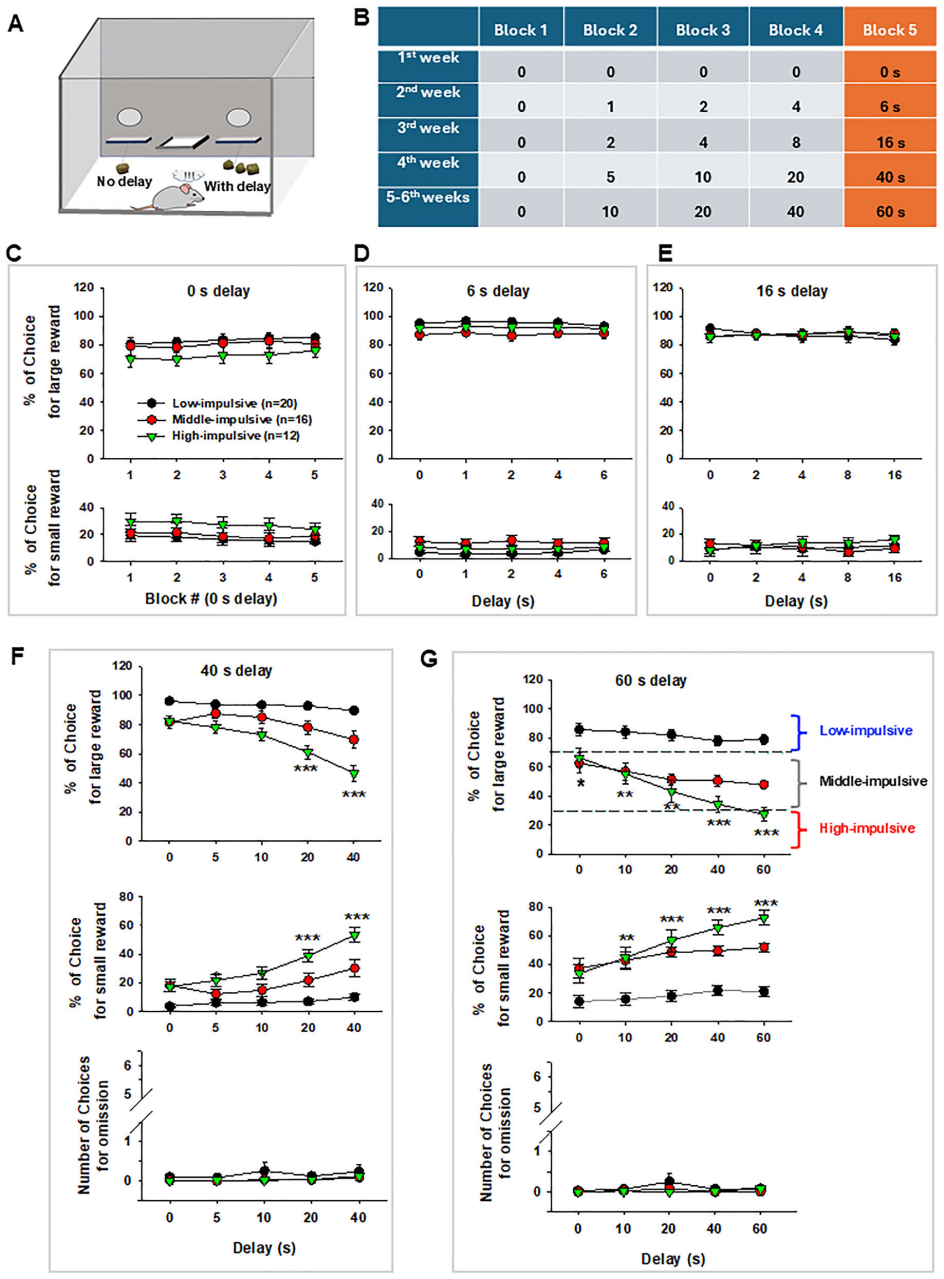
Experimental methods of DDT to identify low-, middle-, and high-impulsive rats. ***A***, A diagram illustrating the DDT for small immediate rewards versus larger delayed rewards. B, Delay schedules during 6 weeks of reward DDT training following an initial 2 weeks of food self-administration (SA) training. ***C–G***, The DDT functional curves, assessed by the percentage of choices for large versus small rewards in low-, middle-, and high-impulsive rats. The data for each delay represents the averaged value over the last 5 sessions (days) at 0 s (***C***), 6 s (***D***), 16 s (***E***), 40 s (***F***), and 60 s (***G***) terminal delay. No significant omissions were observed during the 6 weeks of DDT training (***F***, ***G***). **p < 0.01; ***p < 0.001, compared with low-impulsive rats.

The DDT procedures are the same as we reported recently (Shen et al., 2024). Briefly, the behavioral experiments included 2 weeks of initial food SA training under fixed-ratio 1 (FR1) reinforcement, and 6 weeks of DDT with different terminal delays (30 sessions; Fig. 1).

##### Food SA training

Rats were initially trained on a daily 1 h FR1 schedule for 2 weeks until reliable response was achieved. A rodent diet food pellet (LabTab Ain-76A, TestDiet) served as a reinforcer. Each pellet is 45 mg and contains 5.1% fat, 65.2% carbohydrate, 4.8% fiber, and 2.9% ash contents. For each session, both response levers extended into the chamber. The light cue-paired “active” lever delivered 1 pellet per press and the “inactive” lever failed to elicit cues or reward delivery. The location of the active lever alternated by day. Sessions were terminated upon reaching the pellet delivery maximum (60 pellets) or after 1 h had elapsed.

##### Delay-discounting task

We used a food reward-based DDT adapted from those previously reported (Evenden and Ryan, 1996; Mendez et al., 2010; Xie et al., 2012) to categorize rats as low-, middle, and high-impulsive. After 2 weeks of initial food SA training under FR1 reinforcement, animals were switched to 6 weeks of DDT (30 sessions, 5 sessions per week, 5 blocks per session; Fig. 1). Each block had two forced-choice trials and six free-choice trials for a total of 40 trials/session.

Forced-choice trials presented subjects with one lever per trial, with one trial for small reward and another trial for large reward. The purpose of forced-choice trials was to reinforce the reward size associations for each lever; thus, in these trials the large reward lever delivered three pellets without a time delay. In six free-choice trials, both small and large reward levers were extended into the chamber. Choices for small reward, large reward, or omission (e.g., no lever response on either small reward or large reward) were made within the initial 30 s in each trial. Lever presses were paired with light and sound cues. Trial lengths were such that if the rat pressed the small reward lever, the next trial did not begin until the delay period of the large reward lever elapsed. This ensured that the overall session length was the same for all rats. The location of the large reward lever was counterbalanced across groups and remained consistent for each subject across all experiment sessions.

After six free-choice trials, the next block began and the delay to large reward delivery was increased within-session across consecutive blocks (Fig. 1*C–G*). As the delay increased, preference shifted from the large reward to the smaller, more immediate reward. Animals were exposed to five different delay series, with the terminal delay gradually increasing on a weekly basis. Once animals responded reliably at the 0 s delay with a large reward choice of 80% or greater (five of six trials) for at least five consecutive sessions (Fig. 1*C*), they were placed on the next delay series for a higher terminal delay value. Animals were exposed to each terminal delay for a minimum of five sessions.

After the completion of 30 DDT sessions, animals were classified into three groups based on their stable DDT behavior, assessed by the percentage of choices for large rewards at the 60 s delay, after removing any omission choices from the total choices. Low-impulsive rats were defined as those making ≥70% of choices for the large reward for at least 3 consecutive sessions (days). High-impulsive rats were defined as those making <30% of choices for the large reward for at least 3 consecutive sessions (days; Fig. 1*G*). Other rats making 30–70% of choices for large reward were defined as middle-impulsive rats. The proportion (%) of choices for the larger, delayed reward at each terminal delay block (5th block) across 5 d (sessions) was used to generate the DDT curve. The upward or downward shift of the curve was used to evaluate the effects of cocaine SA on DDT choice decision-making.

### Experiment 2: resting-state fMRI

#### Animal preparation

The procedures of resting-state fMRI for studying functional connectivity were the same as reported previously (Fredriksson et al., 2021). Briefly, rats were anesthetized with isoflurane (2.5%) in oxygen enriched air (70% N_2 _+ 30% O_2_). A bolus dose of dexmedetomidine (0.015 mg/kg, i.p.) was injected 10 min after introduction of isoflurane. Following injection, rats were transferred to a customized MRI-compatible holder with ear bars for head fixation. Dexmedetomidine was administered continuously (0.015 mg/kg/h) through subcutaneous infusion with an infusion pump (PHD 2000, Harvard Apparatus). Isoflurane was lowered and maintained to 0.5–0.75% (0.5% step size for every 5–10 min). Blood oxygenation level-dependent (BOLD) fMRI recording occurred at least 90 min after initial anesthesia. Continuous arterial blood oxygen saturation level and heart rate (MouseOx Pulse Oximeter, STARR Life Sciences) and respiration rate (Small Animal Monitoring and Gating System, SA Instruments) were monitored throughout the imaging session to ensure physiological stability and animal safety (Lu et al., 2012). Rats were maintained at 37 ± 1°C body temperature by a temperature-controlled water-heating pad. Under the combination of dexmedetomidine infusion (0.015 mg/kg/h) and low isoflurane concentration (0.5–0.75%) anesthetic regime, rat respiration rate is ∼60 breaths per minute.

#### Image acquisition

MRI data were acquired using a Bruker BioSpin 9.4 Tesla scanner (Bruker Medizintechnik) with a birdcage coil for RF excitation and single-loop surface coil for signal reception. One high-resolution T2-weighted structural image was collected using rapid acquisition with relaxation enhancement sequence with the following parameters: repetition time (TR), 3,100 ms; echo time (TE), 36 ms; slice thickness, 0.6 mm; slice gap, 0.1 mm; slice number, 31; field of view (FOV), 30 × 30 cm^2^; in-plane matrix size, 256 × 256. Three 10 min resting-state fMRI scans with reverse phase encoding directions were collected using gradient echo echo-planner imaging sequence with the following parameters: TR, 1,500 ms; TE, 15 ms; slice thickness, 0.6 mm; slice gap, 0.1 mm; slice number, 19; FOV, 30 × 30 cm^2^; in-plane matrix size, 80 × 80. For cerebral blood volume (CBV) MRI measurements, monocrystalline iron oxide nanoparticles (MION; Feraheme, AMAG Pharmaceuticals, ferumoxytol 510 mg/17 ml) was injected as contrast agent via a tail vein catheter at a dose of 15 mg/kg. A precontrast multi-gradient echo (MGE) sequence was acquired with the following parameters: TR, 600 ms; TE, 2 ms; FOV, 30 × 30; in-plane matrix size, 128 × 128; slice thickness, 0.765 mm; slice number, 17; echo number, 10; echo space, 2 ms. Two minutes after MION injection, a postcontrast MGE scan was then collected.

### Experiment 3: cocaine SA

#### Surgery

Rats used in cocaine SA experiments were implanted intravenously (i.v.) with a microrenathane catheter (Braintree Scientific). Each rat was anesthetized first with xylazine/ketamine (10/100 mg/kg, i.p.), and a small incision was made to the right of the midline of the neck to expose the external jugular vein. One end of the intravenous catheter was inserted into the vein with the catheter tip reaching the right atrium. The catheter was then secured to the vein with silk suture and the other end fed subcutaneously around the back of the neck to exit near the back of the skull, connected to a bent 24 gauge stainless steel cannula (Plastics One). The catheter and the guide cannula were secured to on the back. The incision was then sutured. Rats were allowed at least 5 d of recovery before SA training. The catheters were flushed every 24–48 h throughout the experiment with gentamicin (Butler Schein; 5 mg/ml) and sterile saline.

#### Procedure

Cocaine SA sessions were conducted in standard operant conditioning chambers (Med Associates) housed in a sound-attenuating box. Each chamber was equipped with two retractable levers, a white light above the active lever and a drug line connected to a Razel syringe pump (Razel Scientific Instruments) set at 3.33 rpm. Rats were trained to lever press for cocaine (0.75 mg/kg/infusion) under a FR1 schedule of reinforcement during daily 3 h sessions. Active lever press resulted in intravenous delivery of cocaine and exposure to a drug-paired cue light and 2,900 Hz sound cue that lasted for the duration of the infusion. Inactive lever responses were recorded but did not evoke cue or drug delivery. To prevent drug overdose, each animal was limited to a maximum of 60 infusions per SA session. To determine whether prolonged cocaine SA alters impulsive choice, each animal was trained for cocaine SA under FR1 reinforcement for 5 weeks (5 d per week).

**Figure 2.**
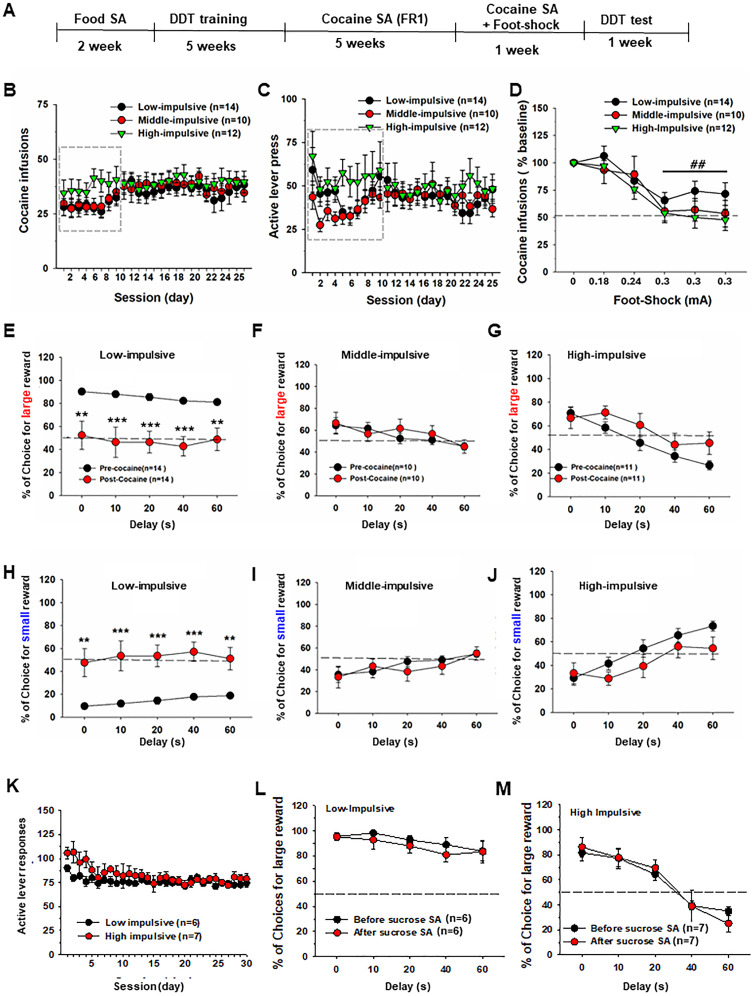
Cocaine SA in low-, middle-, and high-impulsive rats and its effects on impulsive choice decision-making. ***A***, Timeline of the experimental procedures. ***B***, ***C***, The three groups of rats exhibited equivalent levels of cocaine SA, with no between-group differences in either the total number of cocaine infusions (***B***) or active lever responses (***C***) under the free-access (FR1) reinforcement schedule. ***D***, Intravenous cocaine SA (infusions) in the presence of electrical footshock in the three groups of rats, showing that electrical footshock produced a significant reduction in cocaine SA in a current intensity-dependent manner (^##^*p *< 0.001, compared with baseline at the 0 mA current). However, there were no differences in cocaine SA between the three groups. ***E–G***, Impulsive states of low- (***E***), middle- (***F***), and high-impulsive (***G***) rats before and after cocaine SA, showing that cocaine SA significantly decreased the choices for a large reward and shifted the delay–response curve downward only in low-impulsive rats (***E***), indicating an increase in impulsive behavior. ***H–J***, Effects of cocaine SA on impulsive choices in three groups of rats. Cocaine SA caused an increase in the choices for small, immediate rewards only in low-impulsive rats (***H***), indicating an increase in impulsive behavior. ***K***, Oral sucrose SA in low- and high-impulsive rats. There were no differences in the total number of active lever responses (***K***) between the two groups. ***L***, ***M***, Oral sucrose SA did not alter impulsive choice in either low-impulsive (***L***) or high-impulsive rats (***M***) (**p < 0.01, ***p < 0.001, compared with pre-cocaine SA).

Following 25 d of cocaine SA, a 5 d punishment phase was introduced to determine whether high- and low-impulsive rats displayed different drug-taking behavior in the presence of footshock punishment. During the test, a pseudorandom 0.5 s footshock accompanied cocaine SA on half of the reinforced lever presses. The intensity of footshock varied over the 5 d in a predetermined, fixed order (0.18, 0.24, 0.3, 0.3, and 0.3 mA; Fig. 2*D*).

After the compulsive cocaine SA test, the animals were re-evaluated for impulsive choice behavior with five sessions of DDT. Each daily session consisted of five blocks with five different terminal delays from 0 to 60 s. The impulsivity level in each animal was determined by the performance (% of choices for large reward) at the last 60 s delay block.

### Experiment 4: sucrose SA

As a control measure for cocaine SA experiments, oral sucrose SA was evaluated for alterations in impulsive choice behavior. Procedures for oral sucrose SA in rats were the same as we reported previously (Bi et al., 2020). Briefly, rats were trained to self-administer sucrose for 6 weeks under an FR1 schedule of reinforcement during daily 1 h sessions. Active lever responses delivered 0.1 ml 5% liquid sucrose onto a liquid food receptacle paired with light and sound cues. Inactive lever responses were recorded but did not evoke cue or sucrose delivery. During the 4.2 s sucrose delivery period, active lever responses were recorded but did not lead to additional reward delivery. Each animal was limited to a maximum of 60 infusions per SA session, which was the same as in cocaine SA training. After 6 weeks of sucrose SA, rats underwent DDT testing for 1 week as described above.

### Experiment 5: yoked saline “SA”

Saline “SA” was conducted in standard operant chambers (Med Associates). After a 1 week recovery period following catheterization surgery, each group (high- vs low-) of animals was divided into two subgroups: cocaine SA rats (leader rats) and saline SA rats (yoked rats). Leader rats were trained to self-administer cocaine as described above (0.75 mg/kg/infusion, 3 h per day, for 25 d on an FR1 reinforcement schedule). Each leader rat was paired with two yoked rats, which received saline infusions of equivalent volume whenever the leader rat received a cocaine infusion.

### Experiment 6: open-field locomotion

This experiment was designed to compare locomotor response to acute cocaine challenge between high- and low-impulsive rats with or without a history of cocaine SA. Control rats (without cocaine SA) or cocaine SA rats were placed in locomotor detection chambers (Accuscan) and habituated for 1 h. Each rat randomly received vehicle or one dose of cocaine (10 mg/kg, i.p.). Following injection, locomotor activity was recorded for 2 h in 10 min intervals. Each animal was tested two times for vehicle and cocaine in a counterbalanced manner. The time interval was 2–3 d between the tests. The distance counts (cm) were used to evaluate the effects of cocaine on locomotion.

### Experiment 7: RNAscope in situ hybridization

RNAscope multiplex fluorescent assays (Advanced Cell Diagnostics) were well-characterized approaches to detect specific gene transcript expression in the brain (Wang et al., 2012). Whole brains were rapidly harvested from isoflurane-anesthetized rats and frozen in dry ice-cooled methylbutane. Brain tissue was sectioned coronally at 14 μm in a −18°C cryostat (Leica CM3050S) followed by immediate mounting onto Fisher Scientific SuperFrost Plus Slides. In situ hybridization assay procedures were performed according to manufacturer protocols. The following probes (purchased from Advanced Cell Diagnostics) were used to detect *Drd1*, *Drd2*, and *Drd3* mRNA expression: *Drd1* [catalog #895071-C3, *Rattus norvegicus* dopamine receptor D_1_ (*Drd1*) mRNA], *Drd2* [catalog #315641-C2, *Rattus norvegicus* dopamine receptor D_2_ (*Drd2*), mRNA], and *Drd3* [catalog #449961, NAscope Probe - Rn-*Drd3* - *Rattus norvegicus* dopamine receptor D_3_ (*Drd3*) mRNA]. Slides were coverslipped with fluorescent mounting medium (ProLong Gold Antifade reagent P36930; Life Technologies) and imaged with an Olympus FluoView FV1000 confocal microscope at 40× magnification using manufacturer-provided software (Olympus Fluoview FV1000, Olympus). Cells expressing D_1_, D_2_, and D_3_ mRNA in the prelimbic cortex, infralimbic cortex, insula, nucleus accumbens core (NAc-core), and shell (NAc-shell) were counted in a double-blind manner from three stereologically selected sections per brain, with 100 mm rostral-caudal spacing between sections (Shen et al., 2018, 2024). All images were taken under identical optical conditions. Numbers of D_1_-, D_2_-, D_3_-, and DAPI-positive cells were counted using ImageJ cell counter function. Cells were considered DA receptor positive if there are ≥5 puncta clustered around the cell nucleus stained by DAPI (Shen et al., 2024). The thresholding was kept consistent across images.

### Statistical analyses

#### Behavioral data

Data are presented in figures as means (±SEM). Animal group sizes were chosen based on a power analysis (*n* ≥ 7 per group) and extensive previous experience with the animal behavioral models used. The group size is the number of independent values (individual animals), and statistical analysis was performed using these independent values. The investigators were blinded to the group allocation during the experiments and when assessing the outcome. Delay-discounting data from individual subjects were grouped by terminal delay value. To show baseline discounting functions and cocaine SA-induced shifts, group discounting functions are presented at each terminal delay. A rightward shift in the discounting curve following cocaine SA was considered to be an increasing preference for large reward and decrease in impulsive choice. One-way ANOVA was used to examine the differences in DA receptor expression between groups. Two-way repeated-measures ANOVA were used to evaluate the DDT and locomotor behaviors between different groups. Post hoc comparisons consisted of a Student–Newman *t* test (*p *< 0.05).

#### fMRI data

All CBV-based MGE data were preprocessed using an in-house MATLAB based processing pipeline (Lu et al., 2004; Ma et al., 2024). Since MION was injected as an intravascular contrast agent, the changes in the transverse-relaxation-rate pre- and post-MION injection $\left(\Delta R_{2}^{*}\right)$ are proportional to voxel-wise CBV (Mandeville et al., 1998):

$$\mathrm{CBV}\;\propto \;\Delta R_{2}^{*},$$

We then carried out a voxel-wise group (low-impulsive, high-impulsive) × impulsivity index (as a quantitative variable) linear mixed-effect ANCOVA to assess the differential relationships between CBV levels and impulsivity levels in the two groups before and after cocaine SA. Impulsivity index was calculated based on the percentage choice for large reward over all choices. All voxel-wise statistical results were corrected for multiple comparisons using Monte Carlos simulation in AFNI (uncorrected *p *< 0.05, corrected *p *< 0.05, cluster size > 31). We then extracted CBV values for clusters showing significant interactions for further analyses.

All resting-state fMRI data were preprocessed with a conventional pipeline using Analysis of Functional NeuroImages (AFNI; Cox, 1996), Advanced Normalization Tools (ANTs; http://stnava.github.io/ANTs/; Avants et al., 2011), and FMRIB Software Library (FSL; https://fsl.fmrib.ox.ac.uk), including procedures of distortion correction (Smith et al., 2004), skull stripping, motion correction, coregistration to a template, noise component removal, bandpass filtering (0.01–0.1 Hz), and spatial smoothing (FWHM = 0.8 mm). We then conducted seed-based functional connectivity analysis on preprocessed resting-state fMRI data. Three significant brain regions derived from the above voxel-wise CBV results were used as seed regions, including midbrain, thalamus, and auditory cortex. For each resting-state fMRI scan, the seed-based functional connectivity maps were generated by calculating the whole-brain voxel-wise Pearson's correlation coefficients with time course of the seeds. The correlation values were then converted to *z*-scores to achieve normal distribution (Tsai et al., 2020; Fredriksson et al., 2021). Subject-level connectivity maps were then generated by averaging the results from three resting-state fMRI scans.

We then conducted a voxel-wise group (low-impulsive, high-impulsive) × impulsivity index (as a quantitative variable) linear mixed-effect ANCOVA to assess the differential relationships between functional connectivity and impulsivity levels of the three brain regions (midbrain, thalamus, and auditory cortex) of high- and low-impulsive rats before and after cocaine SA. All seed-based statistical results were corrected for multiple comparisons and used corrected *p *< 0.05 (with uncorrected *p *< 0.05 and cluster size > 86 using Monte Carlo simulation in AFNI) as a threshold to determine significant brain voxels. Functional connectivity signals from clusters showing significant interactions were then extracted for further analyses.

## Results

### Identifying subgroups of rats based on their distinct impulsive choices in DDT

Using the criteria described above and shown in Figure 1*G*, we classified 12 out of 48 DDT-trained rats as high-impulsive, 20 rats as low-impulsive, and 16 rats as middle-impulsive. When terminal delays were set at 0, 6, and 16 s, the three groups of rats exhibited similar behavioral patterns, showing a preference for the larger reward (Fig. 1*C–E*). However, as terminal delays increased to 40 s, the three groups of rats began to diverge in their responses: high-impulsive rats took fewer large rewards and more small reward, while low-impulsive rats maintained their preference for the larger reward (Fig. 1*F*). A two-way RM ANOVA revealed a significant main effect of impulsivity (Fig. 1*F* for large reward, *F*_(1,30)_ = 66.5, *p *< 0.001), a main effect of delay (*F*_(4,120)_ = 32.1, *p *< 0.001), and a significant interaction between impulsivity and delay (*F*_(4,120)_ = 17.5, *p *< 0.001). The same assays for choices for small reward at 40 s terminal delay (Fig. 1*F*, middle panel) revealed a significant main effect of impulsivity (*F*_(1,30)_ = 66.5, *p *< 0.001), a main effect of delay (*F*_(4,120)_ = 32.1, *p *< 0.001), and a significant interaction between impulsivity and delay (*F*_(4,120)_ = 17.5, *p *< 0.001).

With a further increase in terminal delay to 60 s, high-impulsive rats shifted choices—decreasing their choices for the large reward while increasing their choice for small reward, whereas low-impulsive rats showed no significant change in their preference (Fig. 1*G*). A two-way RM ANOVA revealed a significant main effect of impulsivity phenotype (Fig. 1*G* for large reward; *F*_(1,30)_ = 35.5, *p *< 0.001), a main effect of delay (*F*_(4,120)_ = 23.9, *p *< 0.001), and a significant interaction between phenotype and delay (*F*_(4,120)_ = 10.5, *p *< 0.001). The same assays for choices for small reward at 60 s terminal delay (Fig. 1*G*, middle panel) revealed a significant main effect of impulsivity (*F*_(1,30)_ = 38.1, *p *< 0.001), a main effect of delay (*F*_(4,120)_ = 48.8, *p *< 0.001), and a significant interaction between impulsivity and delay (*F*_(4,120)_ = 12.7, *p *< 0.001). Notably, middle- and high-impulsive rats exhibited a significant and unexpected decrease in choices for large rewards at zero delay compared with low-impulsive rats (Fig. 1*G*). While the underlying reasons for this reduction remain unclear, it may suggest cognitive impairments, such as decision-making deficits, observed in high-impulsive subjects reported previously (Sakurai et al., 2020). No significant omissions were observed during the 6 weeks of DDT training (Fig. 1*F*,*G*, bottom panels).

### No difference in cocaine SA among the three groups of rats

We then compared cocaine SA among the three groups in the absence or presence of footshock punishment. Figure 2*A* shows the general experimental procedures. Unexpectedly, we did not observe significant differences in cocaine SA under FR1 reinforcement schedule among high-, middle-, and low-impulsive rats during 5 weeks of cocaine SA training. A two-way RM ANOVA of the full dataset (Fig. 2*B*) revealed no significant trait main effect (*F*_(2,33) _= 0.63, *p *> 0.05), a significant time main effect (*F*_(24,792) _= 3.39, *p *< 0.001), and no significant interaction between trait and time (*F*_(48,792) _= 0.72, *p *> 0.05). Since the numbers of cocaine infusions appeared to differ among the three groups during the initial 10 d of cocaine SA training (highlighted in gray box), we reanalyzed the data for this time period. The results showed no significant differences among the groups (trait main effect, *F*_(2,33) _= 1.37, *p *> 0.05; time main effect, *F*_(9,297) _= 3.97, *p *< 0.001; trait × time interaction, *F*_(18,297) _= 0.84, *p *> 0.05).

The same assays of the full dataset (Fig. 2*C*) also revealed no significant trait main effect (*F*_(2,33) _= 0.59, *p *> 0.05), a significant time main effect (*F*_(24,792) _= 1.85, *p *< 0.01), and no significant interaction between trait and time (*F*_(48,792) _= 1.16, *p *> 0.05). Since the numbers of active lever responses appeared to differ among the three groups during the initial 10 d of cocaine SA training, we reanalyzed the data for this time period. The results showed no significant differences among the groups (trait main effect, *F*_(2,33) _= 1.74, *p *> 0.05), a significant time main effect, *F*_(9,297) _= 2.76, *p *< 0.001), and no significant trait × time interaction, *F*_(18,297) _= 0.69, *p *> 0.05).

Next, we assessed the effects of electrical footshock punishment on cocaine SA. We found that electrical footshock significantly decreased cocaine SA in all three subgroups of rats in a shock intensity-dependent manner during 5 consecutive days of testing (Fig. 2*D*), while there was no difference in SA among the high-, middle-, and low-impulsive rats in the presence of footshock. A two-way ANOVA revealed a significant footshock main effect (Fig. 2*D*; *F*_(5,10)_ = 25.35, *p *< 0.001) but did not reveal a significant impulsive main effect (*F*_(2,33)_ = 1.09, *p *> 0.05) or footshock × impulsivity interaction (*F*_(10,165)_ = 0.797, *p *> 0.05).

### Cocaine SA increases impulsivity in low-impulsive rats

We then examined whether cocaine SA altered impulsive choice. We found that cocaine SA caused a significant downward shift in the delay–response curve for large reward in low-impulsive, but not in middle- or high-impulsive rats, suggesting an increase in impulsivity (Fig. 2*E*) compared with that before cocaine SA specifically in low-impulsive subgroup. A two-way RM ANOVA revealed a significant cocaine SA main effect in low-impulsive rats (Fig. 2*E*; *F*_(1,26)_ = 14.66, *p *< 0.001), not in middle-impulsive rats (Fig. 2*F*; *F*_(1,18)_ = 0.14, *p *> 0.05) or high-impulsive rats (Fig. 2*G*; *F*_(1.20)_ = 1.59, *p *> 0.05). Post hoc individual group comparisons revealed significant differences in percentage of choices for large reward between pre- and post-cocaine conditions (*p < 0.05). With the decrease for the choices for large reward, low-impulsive rats alternatively chose more small reward (Fig. 2*H*) without significant omission (data not shown), suggesting increased impulsivity in rats after cocaine SA. This was not observed in middle- or high-impulsive rats (Fig. 2*I*,*J*).

### Sucrose SA does not alter impulsive choice decision-making

As a control, we also examined the effects of oral sucrose SA on impulsive choice under the same experimental conditions. Since the middle-impulsive rats did not exhibit any alteration in either cocaine SA (Fig. 2*B*,*C*) or impulsivity (Fig. 2*F*) following cocaine SA, this group was excluded from this control experiment and subsequent experiments. Consistent with the findings for cocaine SA, no difference in oral sucrose SA was observed between low- and high-impulsive rats (Fig. 2*K*; trait main effect, *F*_(1,11) _= 1.58, *p *> 0.05). However, unlike cocaine SA, sucrose SA did not affect impulsive choice in either group (Fig. 2*L*; sucrose SA main effect, *F*_(1,6) _= 0.34, *p *> 0.05; Fig. 2*M*: sucrose SA main effect, *F*_(1,5) _= 2.45, *p *> 0.05).

### No difference in locomotor responses to acute cocaine between high- and low-impulsive rats

**Figure 3.**
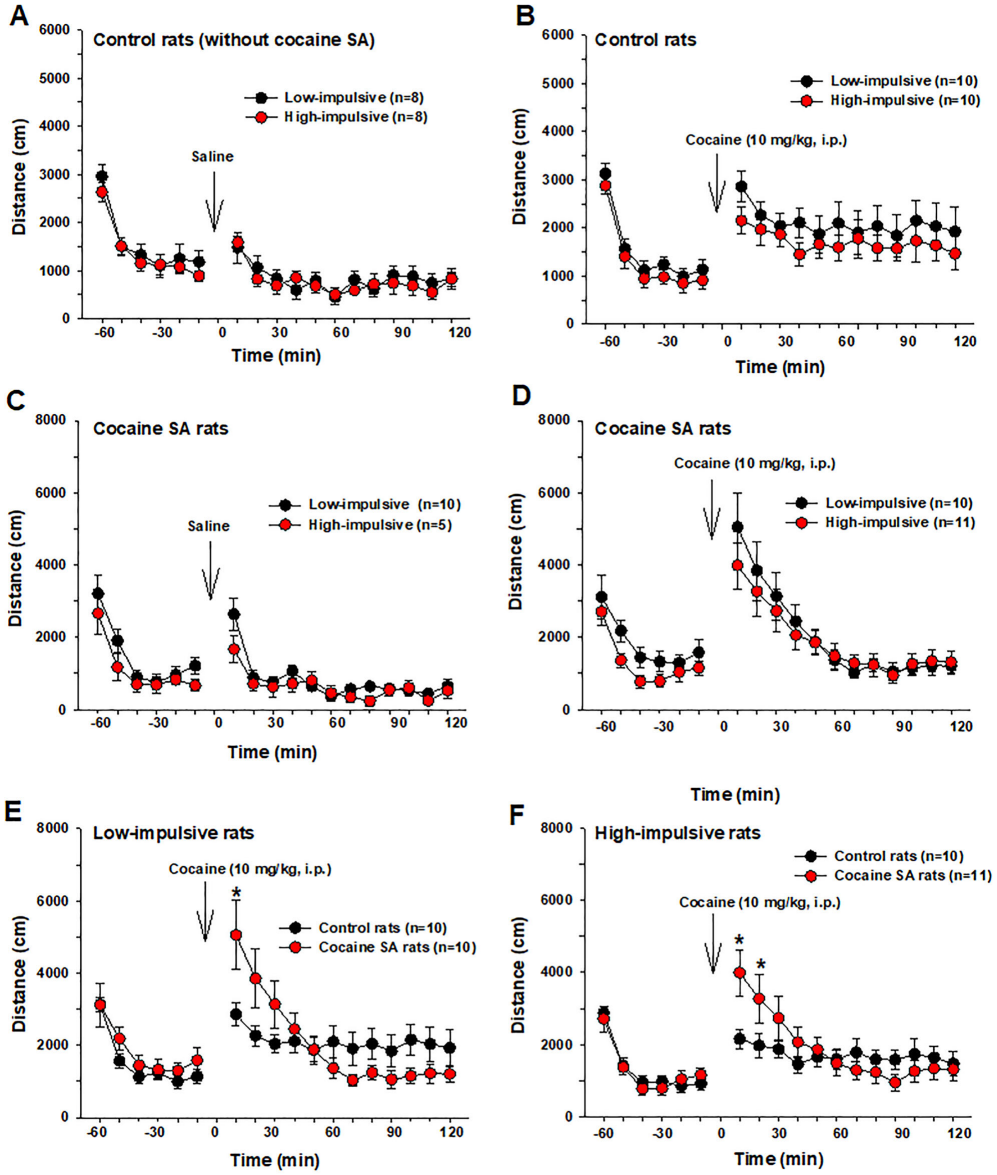
Locomotor response to acute cocaine in two groups of rats with or without a history of cocaine SA. ***A***, ***B***, Locomotor response to saline (***A***) or 10 mg/kg of cocaine (***B***) in both high- and low-impulsive rats without cocaine SA training. ***C***, ***D***, Locomotor response to saline (***A***) or 10 mg/kg of cocaine (***B***) in both high- and low-impulsive rats after cocaine SA training. There is no difference between two groups of rats. ***E***, ***F***, Locomotor response to cocaine in low-impulsive (***E***) and high-impulsive rats (***F***) in rats with or without cocaine SA, illustrating that chronic cocaine SA led to locomotor sensitization to cocaine. *p < 0.05, compared with rats without cocaine SA. The final baseline and all post-injection time point data were included in the statistical analysis.

We also examined open-field locomotor responses to acute cocaine to determine whether high-impulsive rats exhibit heightened locomotor responses to psychostimulants and whether chronic cocaine SA alters these responses. Figure 3 shows that 10 mg/kg of cocaine significantly increased locomotion in rats, regardless of their history of cocaine SA. Notably, there was no difference in locomotor responses to cocaine between high- and low-impulsive rats (Fig. 3*B*,*D*). However, chronic cocaine SA increased locomotor responses to acute cocaine in both groups (Fig. 3*E*,*F*), indicating that chronic cocaine SA led to locomotor sensitization to cocaine.

Two-way RM ANOVA for the data in Figure 3*A–D* did not reveal a significant main effect of saline or cocaine treatment (see Table 1). Conversely, a two-way RM ANOVA for the data in Figure 3*E*,*F* showed a significant main effect of time and a cocaine treatment × time interaction (see Table 1 for details). Post hoc comparisons revealed significant differences at the 10 and 20 min time points following cocaine injection (Fig. 3*E*,*F*; *p < 0.05).

**Table 1.** Statistical results of the locomotor data as shown in Figure 3 by two-way ANOVA

	Treatment main effect	Time main effect	Cocaine × time interaction
Figure 3*A*	*F*_(1,14) _= 0.24, *p *> 0.05	*F*_(12,168) _= 6.11, *p *< 0.001	*F*_(12,168) _= 0.64, *p *> 0.05
Figure 3*B*	*F*_(1,18)_ = 0.93, *p *> 0.05	*F*_(12,216) _= 3.59, *p *< 0.001	*F*_(12,216) _= 0.32, *p *> 0.05
Figure 3*C*	*F*_(1,13) _= 0.75, *p *> 0.05	*F*_(12,156) _= 9.74, *p *< 0.001	*F*_(12,156) _= 1.39, *p *> 0.05
Figure 3*D*	*F*_(1,19) _= 0.16, *p *> 0.05	*F*_(12,228) _= 21.72, *p *< 0.001	*F*_(12,228) _= 0.63, *p *> 0.05
Figure 3*E*	*F*_(1,18) _= 0.01, *p *> 0.05	*F*_(12,216) _= 12.12, *p *< 0.001	*F*_(12,216) _= 5.86, *p *< 0.001
Figure 3*F*	*F*_(1,19) _= 0.28, *p *> 0.05	*F*_(12,228) _= 9.37, *p *< 0.001	*F*_(12,228) _= 4.03, *p *< 0.001

### Impulsivity correlates with regional CBV and functional connectivity in low-impulsive rats

**Figure 4.**
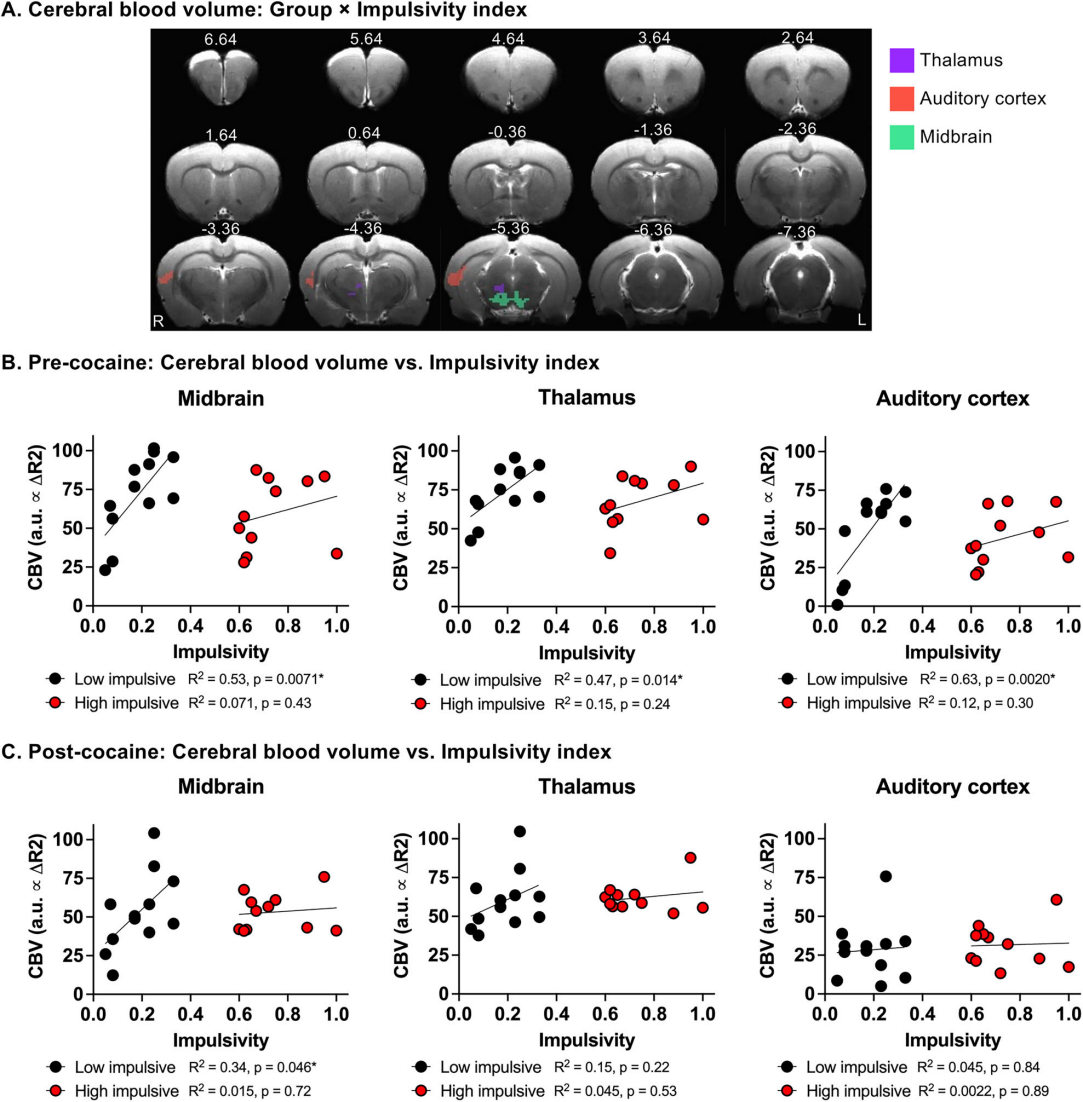
Relationship between impulsivity and regional cerebral blood flow (CBV) in rats before and after chronic cocaine SA. ***A***, Group (Low-impulsive, High-impulsive) × Impulsivity index (as a quantitative variable) interaction of regional CBV levels. Corrected *p *< 0.05. ***B***, ***C***, Relationship between regional CBV levels and impulsivity levels in midbrain (left), thalamus (middle), and auditory cortex (right) before (***B***) and (***C***) after cocaine SA in each individual rats.

To investigate the brain–behavior relationship underlying impulsivity, we conducted a data-driven whole-brain voxel-wise CBV analysis. Linear mixed ANCOVA revealed a significant interaction between group (low-impulsive, high impulsive) and impulsivity index (as a quantitative variable) in regional CBV levels in the midbrain, thalamus, and auditory cortex (Fig. 4*A*; voxel-wise *F*-test, corrected *p *< 0.05). Post hoc analysis indicated positive correlation between CBV levels in these three regions and impulsivity in low-impulsive rats (Fig. 4*B*). In contrast, no significant correlation was observed in high-impulsive rats before cocaine SA (Fig. 4*B*).

**Figure 5.**
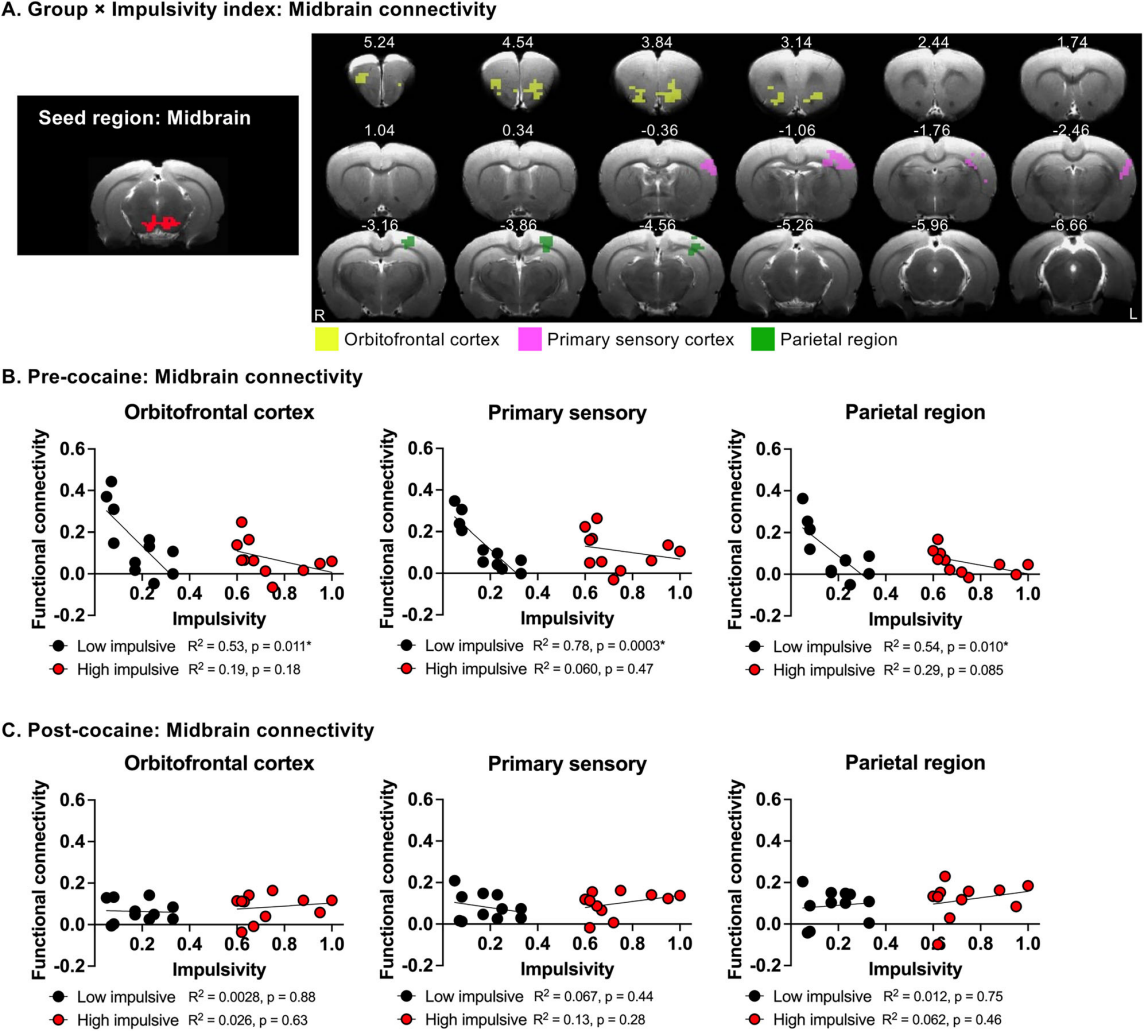
Relationship between impulsivity and functional connectivity of the midbrain seed in rats before and after chronic cocaine SA. ***A***, Left, The midbrain was used as the seed region for functional connectivity analysis. Right, Group (low-impulsive, high-impulsive) × impulsivity index (as a quantitative variable) interaction of voxel-wise functional connectivity of midbrain seed. Corrected *p *< 0.05. ***B***, ***C***, Relationship between midbrain functional connectivity and impulsivity levels between the midbrain and the orbitofrontal cortex (left), primary sensory cortex (middle), or parietal region (right) before (***B***) and after (***C***) cocaine SA in each individual rats.

**Figure 6.**
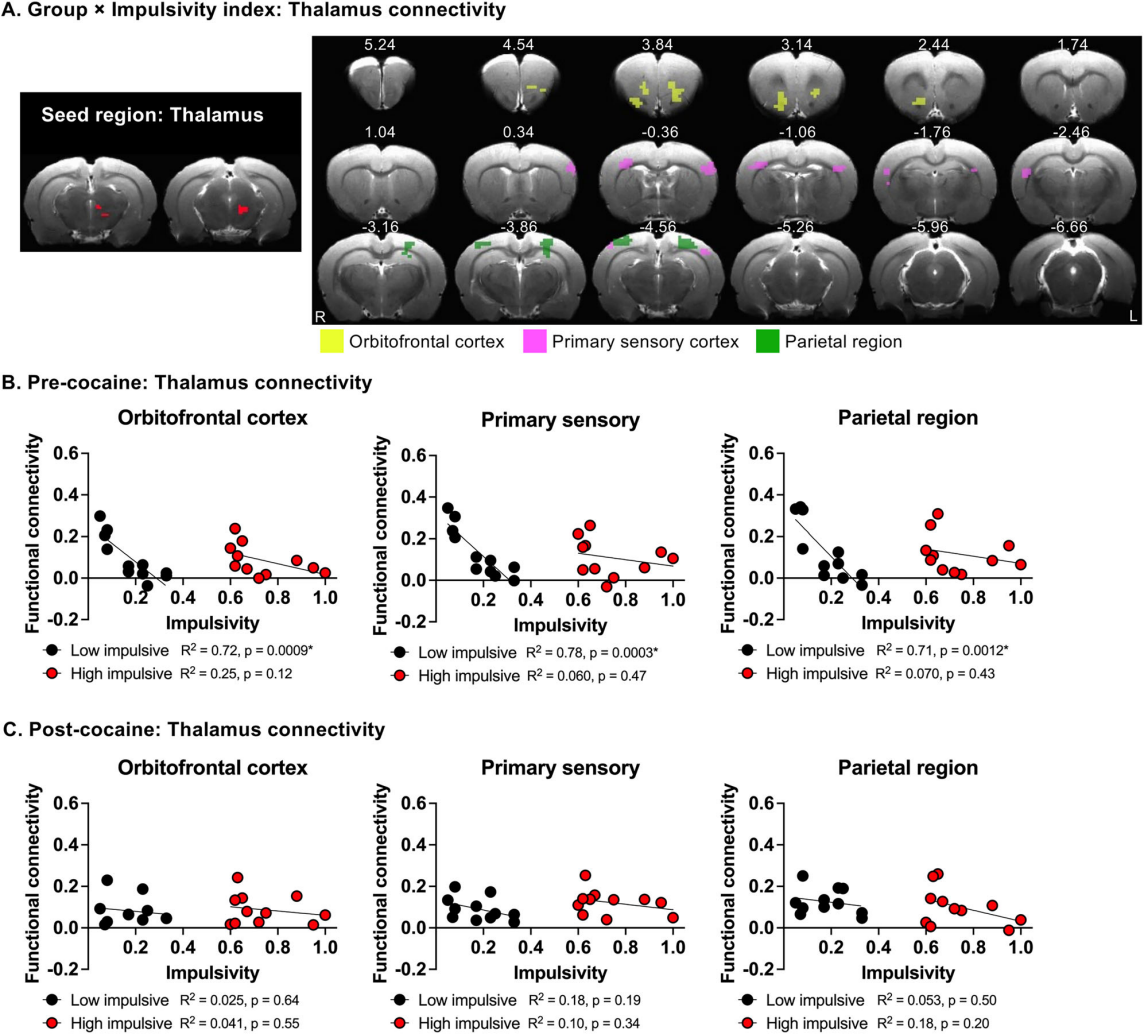
Relationship between impulsivity and functional connectivity of the thalamus seed in rats before and after chronic cocaine SA. ***A***, Left, The thalamus was used as the seed region for functional connectivity analysis. Right, Group (low-impulsive, high-impulsive) × impulsivity index (as a quantitative variable) interaction of voxel-wise functional connectivity of thalamus seed. Corrected *p *< 0.05. ***B***, ***C***, Relationship between functional connectivity and impulsivity levels between the thalamus and the OFC (left), primary sensory cortex (middle), and parietal region (right) before (***B***) and after (***C***) cocaine SA in each individual rats.

To further investigate the neural mechanism underlying impulsive behavior, we conducted a whole-brain voxel-wise functional connectivity analysis using these three CBV-derived brain regions as seeds. Linear mixed ANCOVA revealed a significant interaction between group and impulsivity index (as a quantitative variable) for midbrain functional connectivity with the orbitofrontal cortex (OFC), primary sensory cortex (S1), and parietal region (PTL; Fig. 5*A*; voxel-wise *F*-test, corrected *p *< 0.05). Similarly, the analysis revealed a significant group × impulsivity index interaction (as a quantitative variable) for thalamus functional connectivity with OFC, S1, and PTL (Fig. 6*A*; voxel-wise *F*-test, corrected *p *< 0.05). Post hoc analysis showed a significant negative correlation between the functional connectivity levels of these circuits and impulsivity only in low-impulsive rats (Figs. 5*B*, 6*B*). In contrast, no significant correlation between functional connectivity and impulsivity was found in either midbrain or thalamic circuits in high-impulsive rats (Figs. 5*B*, 6*B*). Additionally, no significant group (low-impulsive, high impulsive) × impulsivity index interaction was found when the auditory cortex was used as the seed region.

### Cocaine SA attenuates the correlation between impulsivity and CBV or functional connectivity in low-impulsive rats

In our DDT experiments, we observed that chronic cocaine SA increased impulsivity level only in low-impulsive rat (Fig. 2). Furthermore, our fMRI experiments demonstrated that impulsivity level positively correlated with regional CBV levels while negatively correlated with functional connectivity also in low-impulsive rats (Figs. 4–6). To further investigate the neural mechanism underlying cocaine-induced changes in impulsivity, we examined post-cocaine CBV levels and functional connectivity in the above identified brain circuits.

Notably, cocaine SA significantly attenuated the positive correlation between impulsivity and regional CBV levels in the thalamus and auditory cortex (Fig. 4*C*). Although the post-cocaine midbrain CBV level remained positively correlated with impulsivity, the correlation coefficient (*r*-value) was reduced (Fig. 4*C*).

In functional connectivity analyses, cocaine SA also significantly attenuated the negative correlation between impulsivity and the functional connectivity of the midbrain with the OFC, S1, and PTL (Fig. 5*C*). A similar attenuation was observed for the functional connectivity of the thalamus with OFC, S1, and PTL (Fig. 6*C*). In contrast, cocaine SA did not significantly alter the correlation between impulsivity and CBV or between impulsivity and functional connectivity in these circuits in high-impulsive rats (Figs. 4*C*, 5*C*, 6*C*).

### Cocaine SA decreased DA receptor expression in corticostriatal brain regions

We recently reported that high-impulsive rats in the DDT exhibit a significant reduction in DA (D_2_, D_3_) receptor expression, with a particular decrease in D_3_R expression, in the NAc (core and shell) (Shen et al., 2024). Notably, restoring D_3_R function by a selective D_3_R agonist significantly relieves or attenuates impulsivity in high-impulsive rats, suggesting that reduced DA receptor expression, particularly D_3_, may play a critical role in impulsive behavior in DDT (Shen et al., 2024). Additionally, our fMRI experiments highlight the significant role of midbrain function—specifically in CBV (regional activity) and functional connectivity (between-region communication)—and its relationship with impulsivity levels. Based on these findings, we hypothesize that cocaine SA may also alter (decrease) DA receptor expression within the mesocorticolimbic DA system, contributing to the decrease in functional connectivity and the increase in impulsivity observed in low-impulsive rats following cocaine SA.

**Figure 7.**
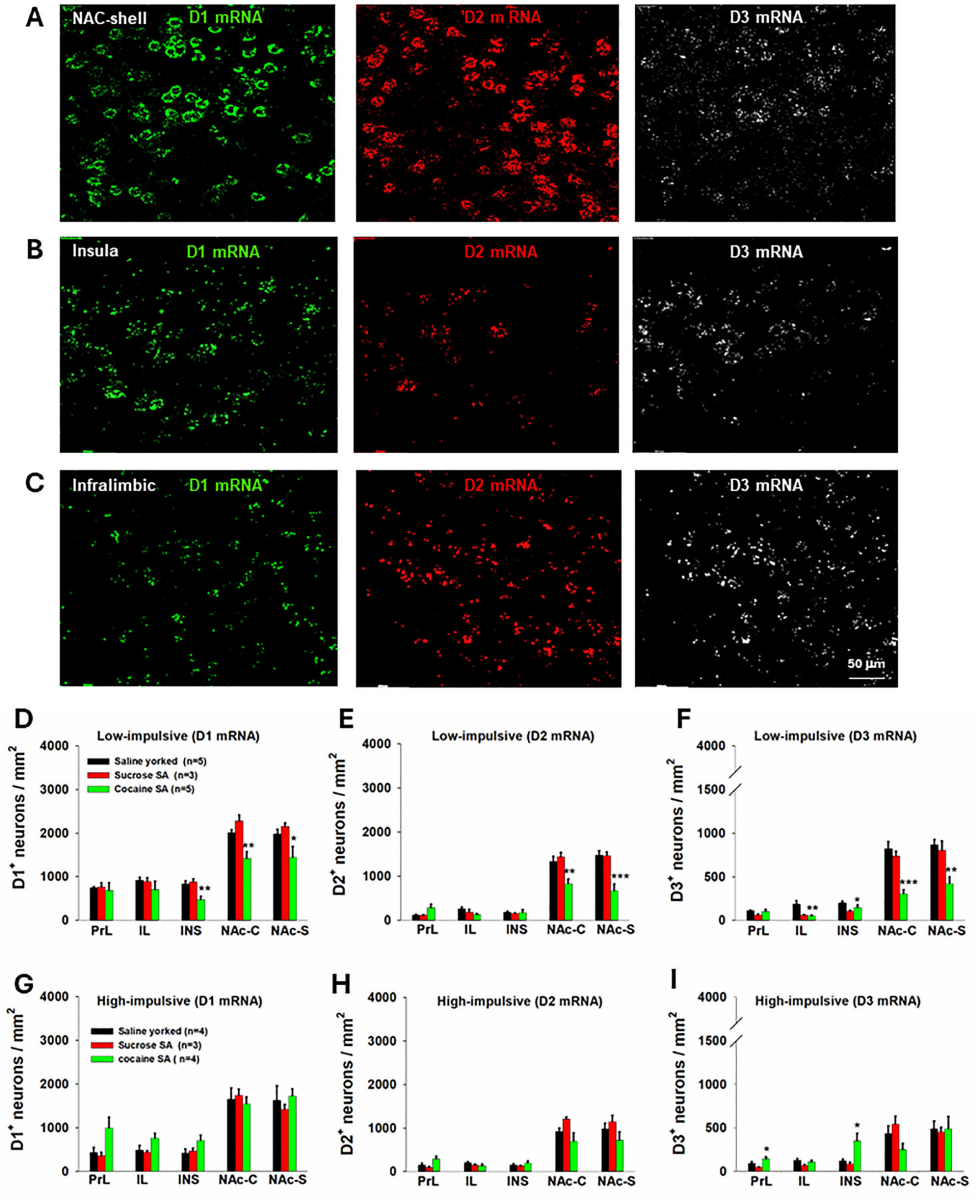
Effects of cocaine SA on brain DA (D_1_, D_2_, and D_3_) receptor mRNA expression in high- versus low-impulsive rats assessed by RNAscope ISH assays. ***A–C***, Representative D_1_ (green), D_2_ (magenta), and D_3_ (gray) mRNA images observed in the NAc-shell (***A***), insula (***B***), and infralimbic cortex (***C***) of low-impulsive rats. ***D–F***, Quantitative cell counting data, showing that cocaine SA significantly decreased NAc D_1_ (***D***), D_2_ (***E***), and D_3_ (***F***) receptor expression in low-impulsive rats. ***G–I***, Quantitative cell counting data, showing that cocaine SA did not significantly alter D_1_ (***G***), D_2_ (***H***), and D_3_ (***I***) receptor expression in high-impulsive rats in most of the regions examined. In contrast, an increase in D_3_ receptor expression was observed in PRL and INS in high-impulsive rats (***I***). *p < 0.05, **p < 0.01, ***p < 0.001, compared with saline or sucrose SA rats.

To test this hypothesis, we used advanced RNAscope ISH to examine the DA receptor mRNA expression (Fig. 7*A–C*) in rats with cocaine or saline/sucrose SA. We found that cocaine SA indeed decreased expression of D_1_, D_2_, and D_3_R in the NAc of low-impulsive (Fig. 7*D–F*), but not high-impulsive, rats (Fig. 7*G–I*). In contrast, a mild increase in D_3_ mRNA expression was observed in the PRL and INS in high-impulsive rats after cocaine SA (Fig. 7*I*). One-way ANOVA revealed a significant reduction in D_1_R expression in INS, NAc-C, and NAc-S (Fig. 7*D*), in D_2_R expression in NAc-C and NAc-S (Fig. 7*E*), and in D_3_R expression in IL, INS, NAc-C, and NAc-S (Fig. 7*F*) in cocaine SA rats in the low-impulsive group compared with yoked saline or oral sucrose SA rats. The detailed statistical results are shown in Table 2.

**Table 2.** Statistical results of the data shown in Figure 7 (RNAscope data) by one-way ANOVA

Region	Figure	Treatment main effects	Figure	Treatment main effect
PrL	Figure 7*D*	*F*_(2,10) _= 0.98, *p* > 0.05	Figure 7*G*	*F*_(2,8) _= 3.74, *p* > 0.05
	Figure 7*E*	*F*_(2,10)_ = 3.24, *p* > 0.05	Figure 7*H*	*F*_(2,8) _= 3.85, *p* > 0.05
	Figure 7*F*	*F*_(2,10) _= 1.72, *p* > 0.05	Figure 7*I*	*F*_(2,8)_ = 5.23, *p* < 0.05
IL	Figure 7*D*	*F*_(2,10) _= 0.76, *p* > 0.05	Figure 7*G*	*F*_(2,8)_= 3.01, *p* > 0.05
	Figure 7*E*	*F*_(2,10) _= 3.31, *p* > 0.05	Figure 7*H*	*F*_(2,8) _= 1.6, *p* > 0.05
	Figure 7*F*	*F*_(2,10) _= 8.88, *p* < 0.01	Figure 7*I*	*F*_(2,8) _= 2.85, *p* > 0.05
INS	Figure 7*D*	*F*_(2,10) _= 10.12, *p* < 0.01	Figure 7*G*	*F*_(2,8) _= 2.43, *p* > 0.05
	Figure 7*E*	*F*_(2,10) _= 0.08, *p* > 0.05	Figure 7*H*	*F*_(2,8) _= 0.74, *p* > 0.05
	Figure 7*F*	*F*_(2,10) _= 4.35, *p* < 0.05	Figure 7*I*	*F*_(2,8) _= 6.29, *p* < 0.05
NAc-C	Figure 7*D*	*F*_(2,10) _= 11.59, *p* < 0.01	Figure 7*G*	*F*_(2,8) _= 0.23, *p* > 0.05
	Figure 7*E*	*F*_(2,10) _= 8.41, *p *< 0.01	Figure 7*H*	*F*_(2,8) _= 3.66, *p *> 0.05
	Figure 7*F*	*F*_(2,10) _= 19.91, *p* < 0.001	Figure 7*I*	*F*_(2,8) _= 3.08, *p* > 0.05
NAc-S	Figure 7*D*	*F*_(2,10) _= 3.46, *p* < 0.05	Figure 7*G*	*F*_(2,8) _= 0.37, *p* > 0.05
	Figure 7*E*	*F*_(2,10) _= 13.77, *p *< 0.001	Figure 7*H*	*F*_(2,8) _= 1.67, *p *> 0.05
	Figure 7*F*	*F*_(2,10) _= 10.75, *p* < 0.01	Figure 7*I*	*F*_(2,8) _= 0.03, *p* > 0.05

## Discussion

In this study, we used DDT to evaluate the causal relationship between choice impulsivity and cocaine SA in rats. We found no difference in free-access cocaine SA or in locomotor response to acute cocaine between high- and low-impulsive rats, indicating that choice impulsivity could not be a risk factor for cocaine use disorder. In contrast, chronic cocaine SA caused a significant increase in impulsivity in low-impulsive rats, but not in middle- and high-impulsive rats, suggesting that cocaine use could be a susceptible factor in developing impulsive behavior in normally low-impulsive subjects. Functional MRI analysis showed that chronic cocaine SA significantly disrupted the correlation between impulsivity and the functional connectivity between the midbrain and the frontal cortices, as well as between the thalamus and the frontal cortices in low-impulsive rats. Further studies showed that cocaine SA also reduced DA (D_1_, D_2_, D_3_) receptor expression in the NAc of low-impulsive rats. These findings suggest that a reduction in DA receptor expressions observed in low-impulsive rats after cocaine SA may be associated with impaired brain functional connectivity in DA-related network and enhanced impulsive behavior.

### DDT choice impulsivity could not be a predictor of cocaine use and abuse

Impulsivity is a personality characteristic associated with behaviors like violence, binge eating, and gambling and is a key feature in disorders such as bipolar disorder, impulse-control disorder, and substance use disorder (Meyer-Lindenberg et al., 2006; First, 2013; Huang et al., 2024; Martinelli et al., 2024). Impulsivity also affects decision-making by leading individuals to overlook negative consequences and take high risks (Bennett et al., 2017; Ioannidis et al., 2019). It is often measured by self-report scales or behavioral tasks, focusing on aspects like response inhibition, delay discounting, and risk-taking (Dalley and Robbins, 2017). However, research on impulsivity faces challenges due to inconsistent definitions, varied measurement tools, and inconclusive findings (Huang et al., 2024).

Our study found that high- and low-impulsive rats, identified by DDT, showed no difference in cocaine SA acquisition and maintenance over 5 weeks under free-access conditions (FR1) or in the presence of electrical footshock. Both groups of rats also failed to show any difference in open-field locomotor response to acute cocaine administration. This finding differs from some previous studies suggesting that high impulsivity predicts cocaine SA escalation in 5CSRT (Dalley et al., 2007; Belin et al., 2008; Broos et al., 2012) and in DDT (Perry et al., 2005; Anker et al., 2009), but it aligns with other reports showing no significant difference in cocaine SA between high- and low-impulsive rats in 5CSRT (Belin et al., 2008; Anker et al., 2009; Caprioli et al., 2013; Abbott et al., 2022), risk choice (Ferland and Winstanley, 2017; Arrondeau et al., 2023), or DDT (Perry et al., 2008). Similarly, conflicting findings were observed in nicotine SA experiments. Motor impulsivity in 5-CSRT was associated with an enhanced motivation for nicotine SA, while choice impulsivity was not in DDT (Diergaarde et al., 2008). Additionally, high-impulsive Roman high-avoidance rats also show mixed results: motor impulsivity predicts higher susceptibility to cocaine-taking and cocaine-seeking, while high-risk impulsive choice does not (Fattore et al., 2009; Dimiziani et al., 2019; Arrondeau et al., 2023). Our findings provide additional evidence indicating that choice impulsivity in DDT does not predict compulsive cocaine-taking behavior. Given the diversity of impulsivity dimensions, it seems that only specific types of impulsivity, such as motor impulsivity, rather than risk or delay-discounting impulsivity, may play a more significant role in predicting substance use and abuse.

### Chronic cocaine exposure enhances impulsive behavior in low-impulsive rats

Another important finding in this study is that chronic cocaine SA selectively increased impulsivity in low-impulsive, but not in high-impulsive, rats. This suggests that chronic cocaine use could be a risk factor for developing impulsive behavior in normally low-impulsive subjects. This finding is consistent with previous reports that chronic cocaine administration (whether experimenter- or self-administered) increases impulsive choice (Paine et al., 2003; Winstanley et al., 2007; Anker et al., 2009; Dandy and Gatch, 2009; Mendez et al., 2010; Zuo et al., 2012; Mitchell et al., 2014b).

Clinical observations also indicate that high-impulsive behavior is often seen in cocaine and other drug users (Lejuez et al., 2005; Moeller et al., 2005; Hanson et al., 2008). However, it was also reported that chronic cocaine or heroin SA failed to alter premature responding (Dalley et al., 2005; Urueña-Méndez et al., 2023) or risky decision-making in male rats (Orsini et al., 2020) or produced a reduction in impulsive action in 5CSRT (Dalley et al., 2007;Caprioli et al., 2013). Similarly, in the risk decision-making task, risk aversion females showed greater cocaine intake under the short-access conditions (Orsini et al., 2020). Together, these findings suggest that chronic cocaine may have different effects on distinct dimensions of impulsivity, increasing impulsive choice in DDT, while having no effect or causing a reduction in impulsive action in 5CSRT.

### Cocaine SA attenuates correlations between impulsivity and brain functional activity in low-impulsive rats

The third important finding of this study is that impulsivity is positively correlated with CBV in the midbrain, thalamus, and auditory cortex only in low-impulsive rats, which is significantly attenuated by chronic cocaine SA. Using these three identified brain regions as seeds, we observed a negative correlation of impulsivity and the functional connectivity between the midbrain and frontal cortices, as well as between the thalamus and frontal cortices, including the OFC, primary cortex, and parietal cortex, which is also attenuated by chronic cocaine SA. This finding suggests that the disruption of the normal functional activity in the midbrain- and thalamus-related networks may underlie chronic cocaine-induced increase in impulsivity observed in low-impulsive rats. It is unknown why impulsivity is positive correlated to the CBV, while negatively correlated to the functional connectivity. Further research is required to understand the neuronal adaptations reflected by CBV and functional connectivity. Notably, our RNAscope experiments revealed a marked reduction in DA receptor (D_1_, D_2_, D_3_) mRNA expression in the corticostriatal regions of low-impulsive rats following cocaine SA. These findings are consistent with the established role of DA in regulating impulsive behavior (Dalley and Roiser, 2012).

OFC is a key frontal region involved in modulating reward expectancies via DA (Takahashi et al., 2011). Pharmacological inactivation of OFC has been shown to significantly reduce the phasic response of DA neurons in the VTA during a DDT (Jo and Mizumori, 2016). These findings support our observation that midbrain-to-OFC functional connectivity is negatively correlated with impulsivity in low-impulsive rats.

In contrast, findings from 5-CSRT task indicate that chemogenetic (Boekhoudt et al., 2017) or optogenetic (Flores-Dourojeanni et al., 2021) activation of DA neurons in the VTA or the substantia nigra pars compacta does not alter impulsivity but instead induces deficits only in attentional performance. This discrepancy may arise from the differences in behavior paradigms used to assess impulsivity, as distinct neural networks and substrates have been proposed and shown to underlie impulsive action versus impulsive choice (Wang et al., 2016; Robbins and Dalley, 2017; Jones et al., 2021).

In our study, chronic cocaine SA attenuated the negative correlation between impulsivity and the functional connectivity of midbrain and thalamic circuits. Impaired functional connectivity between the VTA and regions including thalamus, NAc, amygdala, medial prefrontal cortex, and posterior cingulate cortex has been consistently reported in chronic cocaine users (Gu et al., 2010; Tomasi et al., 2010; Camchong et al., 2014).

Furthermore, human studies have shown that midbrain connectivity with regions like ventral striatum and medial OFC is significantly and negatively associated with self-reported cognitive impulsivity scores in healthy controls. However, this relationship is absent in methamphetamine-dependent participants (Kohno et al., 2016). Together, these findings suggest that chronic cocaine SA may disrupt normal function of the mesocorticalimbic circuits, contributing to changes in impulsive behavior.

### Hypodopaminergic mechanisms may underlie decreased functional connectivity and enhanced impulsivity

A key finding from our RNAscope assay revealed that cocaine SA significantly decreased DA receptor (D_1_, D_2_, and D_3_) expression in the frontal cortex and ventral striatum (NAc) in low-impulsive rats. In contrast, there was a mild increase in D_3_ mRNA in the PrL and INS of high-impulsive rats. These findings suggest a possible intrinsic link between DA transmission, functional connectivity, and impulsive behavior observed in low-impulsive rats. As high-impulsive rats exhibit a reduction in DA D_2_/D_3_ receptor expression and restoration of DA D_3_ receptor function reduced impulsive behavior in high-impulsive rats (Shen et al., 2024), we hypothesized that cocaine-induced decreases in DA receptor expression may be associated with the changes in impulsivity and connectivity in low-impulsive rats. Further research is needed to address the causal relationship between cocaine-induced changes in impulsivity, connectivity, and DA receptor expression.

### Limitations of the current study

There are several limitations in the current study. First, only male rats were used. Second, a daily 3 h session of cocaine SA was used. Previous reports indicate that male and female rats display different cocaine intake under short-access (2 h) and long-access (6 h) conditions (Orsini et al., 2020). Therefore, it is possible that experiments conducted under long-access conditions might yield different results. Third, pharmacological manipulations were not used to evaluate the chronic cocaine-induced changes in functional activity, connectivity, or impulsive behavior, as done in our previous work (Shen et al., 2024). Fourth, fMRI assays in this study were conducted under anesthesia, which may influence the observed correlations between behavior and brain function. Lastly, the study was performed exclusively on adult rats. Considering previous findings that adolescent and adult rats differ in how risky impulsivity predicts cocaine intake (Mitchell et al., 2014a), future research on DDT should include adolescent rats to provide a more comprehensive understanding.

In summary, we systematically investigated the causal relationships between preexisting choice impulsivity and cocaine SA in rats in this study. We found no significant differences in the acquisition and maintenance of cocaine SA or locomotor response to cocaine between high- and low-impulsive rats. However, we observed that chronic cocaine SA increased impulsivity specifically in normally low-impulsive rats. Further investigation into neural mechanisms revealed that chronic cocaine SA also disrupted the normal brain function in the mesocorticolimbic networks and decreased DA receptor expression in low-impulsive rats. These findings suggest that the relationship between impulsivity and SUDs is more complex than previously thought, potentially depending on the type of impulsivity. Our results do not support the view that preexisting choice impulsivity predicts greater drug intake. Instead, chronic cocaine SA increased impulsivity in normally low-impulsive subjects, which is associated with reductions of functional connectivity and DA receptor expression in DA-related networks.

## Synthesis

Reviewing Editor: Sam Golden, The University of Washington Decisions are customarily a result of the Reviewing Editor and the peer reviewers coming together and discussing their recommendations until a consensus is reached. When revisions are invited, a fact-based synthesis statement explaining their decision and outlining what is needed to prepare a revision will be listed below. The following reviewer(s) agreed to reveal their identity: NONE. Note: If this manuscript was transferred from JNeurosci and a decision was made to accept the manuscript without peer review, a brief statement to this effect will instead be what is listed below.

Dear Dr. Yang,

Thank you for submitting your revised manuscript. It has been re-reviewed by the same two experts, who report that the revised manuscript is appropriate for publication.

## Author Response

Author response to the reviewers' comments Synthesis Statement for Author (Required):

Dr. Yang, Your manuscript has been reviewed by two experts in the field. While both reviewers found the content important and of interest, significant limitations were identified that must be addressed prior to publication.

Broadly, reviewers expressed concerns regarding the interpretation of fMRI data, which showed decreased connectivity between brain regions associated with impulsivity, and reductions in dopamine receptor mRNA expression. The reviewers noted that these findings lack direct evidence linking connectivity changes to impulsivity shifts, as no pharmacological manipulation was conducted to confirm causality. Further issues include incomplete statistical reporting, unexplained variations in reward preference among impulsivity groups, and a need for within-group comparisons in certain analyses. Additionally, discrepancies in impulsivity definitions and a lack of controls in the RNAscope experiment were highlighted. Despite these limitations, the reviewers believe this study could provide valuable insights into the dynamic relationship between impulsivity and SUDs. Full reviewer comments are found below.

Author Response: We thank the editor(s) and reviewers for their thoughtful and constructive feedback. We are encouraged by the recognition of our study's potential contributions to addiction research. Below is a summary of our responses to key issues:

1. Pharmacological MRI: We acknowledge the lack of pharmacological manipulations to establish causality between functional connectivity (FC) changes and impulsivity. This study aimed to explore whether and how chronic cocaine self-administration (SA) alters FC and impulsivity. We have previously demonstrated causal relationships between the changes in dopamine D3 receptor activity and impulsivity (Shen et al., NP, 2024). However, investigating causality between FC changes and impulsivity shifts would require extensive additional studies. To avoid potential impacts of pharmacological manipulation on hemodynamics (and therefore fMRI signal) rather than neuronal activity, we propose and plan to use chemogenetic or optogenetic approaches to manipulate neural circuits and evaluate how manipulation FC alters impulsive behavior.

2. Impulsivity-FC association:

To further address the concern, we re-analyzed behavioral and imaging data. The new analysis results demonstrates that impulsivity is positively correlated with cerebral blood volume (CBV) and negatively correlated with FC in low-impulsive rats (new Figs. 4-6). Chronic cocaine SA disrupted the correlations between impulsivity and CBV or FC only in low-impulsive rats. We believe that our new data at least in part address the reviewer's concern on this issue (causality).

3. RNAscope controls:

Following the reviewers' suggestion, we conducted additional RNAscope experiments using yoked saline and oral sucrose SA groups as controls. Results are included in the revised manuscript (new Fig. 7).

4. Statistical reporting:

The manuscript has been substantially revised. The complete statistical results are now included in the text, Figure legends, or tables.

Reviewer 1:

This group investigated a bidirectional relationship between impulsivity and cocaine self-administration in rats and underlying mechanisms. Using a delay discounting procedure, it was found that rats with different degree of impulsivity show a similar pattern of cocaine self-administration, in the presence or absence of electrical shock. However, low impulsive rats show greater impulsivity after cocaine self-administration. Cocaine self-administration was found to cause changes in connectivity between corticostriatal regions, as indicated by FMRI, and a reduction in dopamine receptor mRNA expression. This study poses interesting questions and uses innovative technologies; however, there are some problems with the interpretation of the results and data analysis.

Author response: We thank the reviewer for the positive comments on the innovative technologies used in our study and the comments on our data analysis and interpretation. Below is our point-by-point response to the specific concerns:

The biggest concern that I have pertains to the FMRI findings and their interpretation.

1. First, it is not clear which of the 20+ changes in connectivity are related to changes in impulsivity. The authors claim that cocaine SA decreased connectivity in low-impulsive rats but there is no evidence that these changes are responsible for changes in impulsivity. In fact, the authors states:" these findings suggest that chronic cocaine SA may disrupt the normal function of frontostriatal circuits, leading to changes in impulsive behavior". There was never manipulation involved and such manipulation is necessary to identify a specific pathway involved in impulsivity.

This is an excellent comment. We agree that there is a lack of causality assays between the changes in connectivity and impulsive behavior. As we stated above, to address this fundamental issue, it would require extensive additional studies. Alternatively, we used a new way to re-analyze the behavioral and fMRI data. The new findings demonstrate that impulsivity is positively correlated with CBV in three brain regions (midbrain, thalamus, and auditory cortex) (new Fig. 4). Then we used these three brain regions as "seeds" to analyze the correlations between the seed-based FC and impulsivity. We found that impulsivity is negatively correlated with FC in midbrain circuits and thalamus circuits, but not in auditory-related circuits in low-impulsive rats (new Figs. 5, 6). Interestingly, chronic cocaine SA disrupted the corrections, particularly in the midbrain- and thalamus-circuits, in low-impulsive rats. Accordingly, we have revised our hypothesis statement about the possible relationship between FC and impulsivity in the revised manuscript (p. 24-25).

2. Similarly, how are changes in DA receptors mRNA expression that are caused by cocaine SA related to changes in impulsivity? DA receptors were never deleted or blocked pharmacologically to make this claim.

We appreciate this excellent point. We have previously demonstrated that high-impulsive rats exhibit reduced D2/D3 receptor expression and that pharmacological manipulation of D3R modulates impulsivity (Shen et al., NP, 2024). Additional studies using D3-KO mice and optogenetics are ongoing that aim to address whether and how pharmacological or transgenic manipulations of DA receptors will alter cocaine-induced changes in impulsivity. We expect that it will take 2-3 years to be completed.

3. It is not clear how cocaine-induced changes in DA receptor mRNA expression are related to loss of connectivity within the corticostriatal network. The authors never explained how these two findings are related, if at all.

We acknowledge that our results lack a direct comparison between cocaine-induced changes in DA receptor mRNA expression and alterations in functional connectivity. This limitation arises because RNAscope ISH experiments are terminal (e.g. after the animals were euthanized), whereas our fMRI experiments are longitudinal and require data collection at two time points from the same rats. Consequently, direct correlations between the two findings could not be made, as separate cohorts of rats were used for each experiment.

Nonetheless, we believe that the two findings complement each other. Cocaine-induced reductions in DA receptor expression, disruptions in midbrain CBV or FC, and increases in impulsivity were consistently observed exclusively in low-impulsive rats. Furthermore, our previous study (Shen et al., 2024) demonstrated a negative correlation between impulsivity and DA D2/3 receptor expression by pharmacological manipulation of DA receptors. To address this concern, we have added two paragraphs to the revised manuscript on pages 24-25 and 30 for further discussion.

4. F-values, degrees of freedom are not reported with the FMRI data and some other experiments, making it difficult to critique these results. In addition, none of the significant interactions were followed by post hoc tests, making the statistical analyses incomplete.

We have re-analyzed all the fMRI data and included complete statistical details in the revised figures (new Figs. 4-6), figure legends, and tables.

5. Findings from oral sucrose administration and locomotor activity experiments were never discussed in the discussion. It is not even clear why these experiments were conducted.

We have added a discussion clarifying that sucrose SA serves as a non-drug reinforcer control for cocaine SA (p. 13), while open-field locomotion assesses acute cocaine effects in low- and high-impulsive rats, in addition to cocaine SA (p. 14). The findings in both cocaine SA and locomotion support a conclusion that both groups of rats exhibit similar behavioral responses to acute cocaine or chronic cocaine self-administration.

6. The RNAscope experiment should include a control group of rats self-administering saline, not naïve rats. Both groups should undergo the same procedures-food restriction, delay discount, surgery, drug/saline self-administration. Otherwise, we are comparing apples to oranges.

This is a fair comment. Accordingly, we conducted additional RNAscope experiments using saline SA and oral sucrose SA rats as controls. The revised Fig. 7 includes these new data, providing a more robust analysis of DA receptor expression changes.

7. In addition, the conclusion regarding RNAscope findings are not complete. It appears that high impulsive rats show a reduction in DA receptor mRNA when compared to low impulsive rats. But this was never analyzed or even mentioned. Also, sample sizes are very small n=3.

This is another excellent comment. The concern regarding the correlation between impulsivity and DA receptor expression has been addressed in our previous report (Shen et al., 2024). We have now cited this reference and discussed it in the revised manuscript (p. 24-25, 30).

Regarding the group size, we should note that larger sample sizes (n=7-8) are often used in behavioral assays due to the inherent variability in behavioral data across subjects. In contrast, smaller sample sizes (n=3-4) are commonly used in RNAscope ISH assays because the variation in mean cell counts (derived from thousands of cells and hundreds of images) across subjects is relatively small, which are sufficient to detect significant changes in RNAscope data. To fully address the reviewer's concern, we have increased the sample sizes for both groups (n=4-5 for cocaine SA groups, n=7-8 for (saline + sucrose) controls).

8. I find it strange that low-impulsive rats before cocaine SA (Fig.2 E-G), prefer larger rewards over smaller rewards at zero delay (90%) but middle and high-impulsive rats just 60%. At zero delay, there should be no difference between low, middle and high impulsive rats. This was never explained.

We appreciate the reviewer's astute observation regarding this unexpected shift in preference for larger rewards at zero delay (Fig. 1G, Fig 2-E, F, G).

While we cannot definitively explain this unexpected observation, we have noted an interesting pattern in our data. As depicted in Fig. 1 (C, D, E), three groups of rats exhibited similar preferences for larger rewards at zero-second delays. However, when the terminal delay increased to 40 or 60 seconds over time (weeks), high-impulsive rats displayed a significant reduction in preference for larger rewards at zero delay (Fig. 1G). This finding suggests that, in addition to higher impulsivity, high-impulsive rats may also exhibit cognitive deficits in the choice decision, which is now discussed in the revised manuscript (p. 18-19).

9. Although the title mentions mesocorticolimbic connectivity, ironically, fMRI study never measured changes in the mesocortical pathway.

To address the reviewer's concerns, we have now re-analyzed the data. As the new imaging data included the alterations in CBV and connectivity in the midbrain-frontal cortex circuits, we kept the original title without any change.

Reviewer 2:

The authors challenge the longstanding view that propensity for impulsive choice is a risk factor for substance use disorders (SUDs). Impulsive choice was tested using a delay-discounting task (DDT), as most studies of the link between SUDs and impulsivity utilized risky choice-based tasks or the 5-choice serial reaction time task, which examines impulsive action. Rats were classified as low, medium, or high impulsivity based on their performance in the task and then subjected to cocaine self-administration. Surprisingly, there was no difference in cocaine self-administration (SA) based on the impulsivity profile, instead impulsivity rats had increased impulsivity in DDT after cocaine SA. Resting-state fMRI showed decreases in functional connectivity before and after cocaine SA in the IL, NAc, and DMS between low- and high- impulsivity rats. RNAscope revealed reveal changes in dopamine receptor expression in these regions. These results suggest that high impulsivity does not predict cocaine use but may result from it. This study made an important contribution to understanding how circuits that mediate impulsivity and SUDs are related.

We thank the reviewer for his/her time, insightful comments, and positive feedback on this manuscript.

Specific feedback:

Content:

In several instances, it may be valuable to perform within-group comparisons:

Fig 1: The analysis compares reward preference between the three impulsivity classifications, there is a lot of variation in the reward preference when the delay is 0s - e.g. in panel C the high- impulsive rats exhibited a 70% preference for the larger reward, in panel D it is 90%, and in panel G it is close to 65%. This is not discussed, however it is not entirely clear that these differences are not significant and the source of the variation is unclear.

We thank Reviewer 2 for raising this insightful question.

As you pointed out above, all three groups of rats exhibited similar preferences for larger rewards at zero-second delays at shorter delays (0, 6, 16, 40 seconds) (Fig. 1-C, D, E, F). However, when the terminal delay increased to 60 seconds after several weeks of DDT training, middle- and high-impulsive rats showed a significant reduction in their preference for larger rewards at zero delay. This finding suggests that, in addition to higher impulsivity, middle- and high-impulsive rats may also exhibit cognitive deficits in decision-making, particularly when faced with choices involving delayed rewards. We have now briefly discussed it and cited references (p. 18-19) to further elaborate on this point.

Fig 3: Active lever responses for cocaine and sucrose between low and high impulsive rats are compared, but post-cocaine DDT responses between groups are not assessed.

The statistical results are now presented in the Results section (p. 19-20).

Fig 7: Locomotor response to acute between high and low impulsive rats before and after cocaine SA is not assessed. It is not clear how long after Cocaine SA the acute cocaine was given.

This experiment aimed to compare the acute locomotor response to cocaine between low- and high-impulsive rats. Our findings indicate no significant differences in locomotor activity between the two groups, consistent with our finding in cocaine self-administration. However, chronic cocaine SA caused locomotor sensitization to cocaine in both groups of rats. This is consistent with literature reports.

To better understand this experiment, we have now moved this figure (new Fig. 3) forward to a location between our behavior finding and fMRI imaging finding.

Other points:

The limitations of using MRI imaging in anesthetized rodents to draw conclusions about circuit activity changes due to cocaine are not discussed.

This is an excellent comment. This limitation is now included in the revised manuscript (p. 30).

The introduction divides impulsivity into choice and action based, with choice split further into risk and delay-discounting. In the discussion, cognitive and motor impulsivity are discussed, which are presumably different terms for choice/action impulsivity. It would be helpful to use a consistent terminology or clarify the two sets of terms explicitly.

We have now used the same terms to avoid confusion in the revised manuscript.

Fig 6: RNAscope was performed solely using dopamine receptor neuron genes, without any control markers to distinguish cell types. This caveat should be discussed and follow up studies could be done in the future.

This is a fair comment. As we highlighted in the revised manuscript (p. 6), this study aimed to determine whether and how cocaine SA alters DA receptor expression. Our next step will be to identify the specific cell types (with specific cell markers), if any, that exhibit changes in DA receptors following cocaine SA. Addressing this question will require extensive work, as DA receptors are expressed in distinct neuronal phenotypes across different brain regions. lines 137, 612 "female rats will be used in our follow up study/research" is not necessary to state.

It is now deleted.

Formatting:

Fig 2A: text labels are off-center and are not aligned.

4A: "anterior cingulate" label is misaligned. line 529 "ignor" should be "ignore".

The typos and errors are now fixed or removed.
